# FDG-PET/CT lymph node staging after neoadjuvant chemotherapy in patients with adenocarcinoma of the esophageal–gastric junction

**DOI:** 10.1007/s00261-016-0820-x

**Published:** 2016-07-12

**Authors:** Pavel Fencl, Otakar Belohlavek, Tomas Harustiak, Milada Zemanova

**Affiliations:** 1 PET Center, Hospital Na Homolce, Roentgenova 2, 150 30 Prague 5, Czech Republic; 2 3rd Clinic of Surgery, First Faculty of Medicine, Charles University in Prague and University Hospital Motol, V Uvalu 84, Praha 5, 150 06 Czech Republic; 3 Clinic of Oncology, First Faculty of Medicine, Charles University in Prague and General Teaching Hospital, U Nemocnice 2, Praha 2, 128 08 Czech Republic

**Keywords:** FDG-PET/CT, Esophageal adenocarcinoma, Nodal staging, Accuracy, Neoadjuvant chemotherapy

## Abstract

**Objectives:**

The aim of the analysis was to assess the accuracy of various FDG-PET/CT parameters in staging lymph nodes after neoadjuvant chemotherapy.

**Methods:**

In this prospective study, 74 patients with adenocarcinoma of the esophageal–gastric junction were examined by FDG-PET/CT in the course of their neoadjuvant chemotherapy given before surgical treatment. Data from the final FDG-PET/CT examinations were compared with the histology from the surgical specimens (gold standard). The accuracy was calculated for four FDG-PET/CT parameters: (1) hypermetabolic nodes, (2) large nodes, (3) large-and-medium large nodes, and (4) hypermetabolic or large nodes.

**Results:**

In 74 patients, a total of 1540 lymph nodes were obtained by surgery, and these were grouped into 287 regions according to topographic origin. Five hundred and two nodes were imaged by FDG-PET/CT and were grouped into these same regions for comparison. In the analysis, (1) hypermetabolic nodes, (2) large nodes, (3) large-and-medium large nodes, and (4) hypermetabolic or large nodes identified metastases in particular regions with sensitivities of 11.6%, 2.9%, 21.7%, and 13.0%, respectively; specificity was 98.6%, 94.5%, 74.8%, and 93.6%, respectively. The best accuracy of 77.7% reached the parameter of hypermetabolic nodes. Accuracy decreased to 62.0% when also smaller nodes (medium-large) were taken for the parameter of metastases.

**Conclusions:**

FDG-PET/CT proved low sensitivity and high specificity. Low sensitivity was based on low detection rate (32.6%) when compared nodes imaged by FDG-PET/CT to nodes found by surgery, and in inability to detect micrometastases. Sensitivity increased when also medium-large LNs were taken for positive, but specificity and accuracy decreased.

There is little doubt that the malignant involvement of lymph nodes is a key factor in the outcome of patients with esophageal carcinoma following radical (R0) resection [1–4]. However, some disagreement remains as to the optimal method for nodal staging in esophageal cancer according to the AJCC scheme [5–7]. Endoscopic ultrasonography (EUS) is currently considered a more sensitive tool than computed tomography [7–9] or contrast-enhanced CT [5] for imaging lymph nodes. Metabolic information from integrated FDG-PET/CT may improve the sensitivity of anatomic evaluation by CT [10, 11], and some authors have reported good results in imaging lymph node metastases using FDG [12–14], but others have not been able to show that such data are helpful [9, 15]. A number of studies have also been performed in patients with squamous cell carcinoma of the esophagus [16] (SCC) or with “esophageal cancer” [17]. These patients have various ratios of SCC and adenocarcinoma (AC) of the esophagus, and as these types of carcinoma differ substantially [2, 18, 19], the results of these studies are only partially applicable to patients with AC. Confronting our FDG-PET/CT data with histologic specimens, we concluded FDG-PET/CT to be of low sensitivity and of high specificity and that is why we tried to verify our opinion using a large cohort of patients using region-based analysis.

## Materials and methods

### Patient participants

One hundred and fifty-three patients with adenocarcinoma of the esophageal–gastric junction were prospectively recruited to the study between January 2009 and February 2013 to ascertain the metabolic response to neoadjuvant chemotherapy. Initial FDG-PET/CT staging was followed by FDG-PET/CT restaging after the first course of therapy and no earlier than 14 days after the third course of neoadjuvant chemotherapy. The data from postchemotherapy examinations were used in the analysis. Seventy-four patients (6 female, 68 male; median age 60 years; range 27–74 years) were surgically resected and were selected for this analysis. The participants were all of middle European origin; of similar socioeconomic background; and without deficits in education, living standard, access to health care, or medical information.

### Therapy

Patients received three preoperative and three postoperative cycles of chemotherapy, with surgery performed 3–6 weeks after completing the third cycle. Intravenous chemotherapy consisted of epirubicin (50 mg/m^2^) and cisplatin (60 mg/m^2^) with hydration and standard antiemetic prophylaxis on day 1 plus continuous infusion of fluorouracil (200 mg/m^2^ daily) for 21 days (ECF) using a portable infusion pump or oral capecitabine (1.000 mg/m^2^ twice daily) for 14 days (ECCap) in a 21 days cycle (according to the MAGIC regimen) [20, 21]. Depending on the localization and extent of the tumor, all patients underwent either transthoracic esophagectomy with gastric pull-up reconstruction and intrathoracic esophagogastric anastomosis (Ivor–Lewis procedure), or proximally extended total or proximal gastrectomy with transhiatal resection of the distal esophagus and suprahiatal anastomosis. All patients that underwent resection had negative proximal resection margins as determined by intraoperative frozen histopathological analysis. Abdominal D1 plus lymphadenectomy (all lymph node tissue around the left gastric artery, common hepatic artery, celiac trunk, and proximal half of the lienal artery) was a standard part of all operations; radical infracarinal mediastinal lymphadenectomy was added to transthoracic esophagectomy.

### Lymph node imaging

Patients fasted for at least 6 h before intravenous administration of FDG. Blood glucose levels were determined in each patient before FDG administration. The dose of FDG was corrected for patient weight as recommended by the European Association of Nuclear Medicine [22]. Examinations were performed without the application of iodinated contrast material intravenously. Patients were positioned in a supine head-first position on the table of a Biograph 40 TruePointTrueV HD PET/CT scanner (Siemens, Erlangen, Germany). For CT data acquisition, the following parameters were used: 120 kV, 63 mAs (effective), collimation 24 × 1.2 mm, and slice thickness 5 mm. Axial images were reconstructed in 2 mm increments using both B19S and B50F kernels. In all examinations, 3 min/bed position, Gaussian filtration, kernel 5 mm, matrix size 168 × 168 were used for PET data acquisition. Using the Siemens TrueX (PSF) reconstruction algorithm, PET data were reconstructed in corrected and noncorrected images, with 2 mm reconstruction increments, three iterations, and 21 subsets. The PERCIST 1.0 protocol was used to maintain reproducibility as described previously [23, 24].

### Surgical sampling of lymph nodes

In 52 out of 74 patients (70.3%) the carcinoma was classified as Siewert type I, in 17 out of 74 patients (23.0%) as Siewert type II, and in 5 out of 74 patients (6.7%) as Siewert type III [25]. Altogether, 1540 lymph nodes were surgically resected and subjected to histological analysis. According to the origin of the specimens, denoted by operating surgeons, topographical regions were identified. Following the principles of lymph node classification [26], lymph nodes were assorted into these five regions: region 1, subcarinal nodes; region 2, periesophageal and preaortal nodes of the lower mediastinum; region 3, left and right pericardial nodes; region 4, perigastric nodes along the lesser curvature and nodes around the left gastric artery; region 5, perivascular nodes. In total, 287 regions (mean 3.8 regions per patient) comprised the matrix used in the analyses [27]. Lymph nodes in every region were counted and histologically categorized as metastatic or nonmetastatic.

### Lymph node diagnosis by FDG-PET/CT

In the design of the prospective study according to the PERCIST 1.0 protocol [23], no more than two of the metastatic lymph nodes were analyzed in each patient. To enable comparison between nodes imaged after the chemotherapy and those surgically removed, examinations were re-evaluated from digitally archived data. The short axis of identified nodes was measured, and FDG consumption was compared with the background. In this way, all nodes were categorized according to the diameter of the short axis either large (>1 cm), small (<0.5 cm), or medium-large (0.5–1 cm), or, according to their metabolic activity, hypermetabolic (FDG consumption that exceeded the consumption in surrounding tissue) [28] or nonhypermetabolic. Next, the nodes were sorted into a matrix identical to that of the surgical specimens. All evaluations were performed by a single radiologist with more than 8 years experience in PET/CT and 20 years in CT diagnostic imaging.

### Comparison between the nodes identified by FDG-PET/CT and the ones surgically resected

Histological evaluation (gold standard) was considered positive when at least one metastatic lymph node was found in a region. FDG-PET/CT evaluation was considered positive when at least one lymph node bore pertinent parameters in the analyzed region. Four FDG-PET/CT parameters were compared to the gold standard: (1) hypermetabolic nodes, (2) large nodes, (3) large-and-medium large nodes, and (4) hypermetabolic or large nodes. Any small and nonhypermetabolic lymph node was considered negative. For statistical analyses, regions where lymph nodes were not imaged by FDG-PET/CT were also classified as negative.

## Results

### Accuracy in the detection of lymph nodes metastases

Only 502 of 1540 surgically resected lymph nodes (32.6%) were also imaged by FDG-PET/CT (Table 1). Comparative data for the four FDG-PET/CT parameters and the gold standard have been tabulated (Table 2). Three of the tested parameters gave similar accuracy: hypermetabolic nodes (77.7%), hypermetabolic/or large nodes (74.8%), and large nodes (72.5%). Despite the fact that the parameter large-and-medium large nodes gave the best sensitivity (21.7%), it proved the worst specificity (74.8%) and accuracy (62.0%), which therefore made it the least reliable for detection of metastases. The other three parameters produced very few false positives with the specificity range from 93.6% to 98.6%.

**Table 1:** Differences in lymph nodes surgically resected and imaged by FDG-PET/CT, divided by regions

	Region 1	Region 2	Region 3	Region4	Region 5	Total
N. resected	293	270	215	334	428	1540
N. imaged	146	91	90	90	85	502
(%)	49.8	33.7	41.9	26.9	19.9	32.6

N. resected: lymph nodes (LNs) surgically resected, N. imgaged: LNs imaged by FDG-PET/CT, Region 1: subcarinal LNs, Region 2: periesophageal LNs of lower mediastinum, Region 3: paracardial LNs, Region 4: perigastric LN, Region 5: perivascular LNs

**Table 2:** Sensitivity, specificity, and accuracy of all four tested parameters

	GS+	GS−	Total		Estimated value	CI95%
(1) Hypermetabolic LNs						
				Sensitivity	11.6	5.49–22.1
Test+	8	4	12	Specificity	98.6	95.7–99.6
Test−	61	214	275	Accuracy	77.7	72.3–82.3
Total	69	218	287	PPV	72.7	39.3–92.7
				NPV	77.9	72.4–92.7
(2) Large LNs						
				Sensitivity	2.9	0.5–11.0
Test+	2	12	14	Specificity	94.5	90.35–97.0
Test−	67	206	273	Accuracy	72.5	66.8–77.5
Total	69	218	287	PPV	14.3	2.5–43.8
				NPV	75.5	69.8–80.3
(3) Medium-and-large LNs						
				Sensitivity	21.7	13.1–33.6
Test+	15	55	70	Specificity	74.8	68.4–80.3
Test−	54	163	217	Accuracy	62.0	56.1–67.6
Total	69	218	287	PPV	21.4	12.9–33.2
				NPV	75.1	68.7–80.6
(4) Hypermetabolic/large LNs						
				Sensitivity	13.0	6.5–23.8
Test+	9	14	23	Specificity	93.6	89.2–96.3
Test−	60	204	264	Accuracy	74.2	68.7–79.1
Total	69	218	287	PPV	39.1	20.5–61.2
				NPV	77.3	71.6–82.1

*GS* gold standard: GS+ regions with metastatic lymph nodes (LNs) found by histology; GS− regions without metastatic LNs; test+ region where at least one LN bears tested parameter; test− negative test; *SE* sensitivity, *PPV* positive predictive value, *NPV* negative predictive value; CI 95%: confidence interval valid for 95% of data

In the analysis, hypermetabolism proved better for detecting metastatic regions than node size. The single metabolic parameter, hypermetabolic nodes, proved positive predictive value of 72.7%, whereas the single anatomic parameter, large nodes, proved positive predictive value of 14.3%. Neither combination of parameters “medium-and-large LNs” nor “hypermetabolic or large nodes” reached the level of metabolic single parameter when showed positive predictive value of 21.4% and of 39.1%, respectively.

### False-negative and false-positive findings

False negatives were primarily due to the inability of FDG-PET/CT to image all lymph nodes found at surgery. Less frequent was the situation when nodes were imaged but not recognized as metastatic due to low metabolic activity or diameter shorter than 1.0 cm [9]. Only once, large lymph nodes adjacent to the tumor mass were differentiated from the tumor itself in region 4.

Four out of 218 nonmetastatic regions (1.8%) were falsely identified as metastatic ones. In three of them, different types of inflammation were found: a granulomatous inflammation with anthracosis in region 1, necrotic LNs with foamy histiocytes along with microscopic ulceration of the tumor in region 4, and histiocytosis in region 5. No histologic explanation could be found for one region [29].

## Discussion

The exclusive selection of patients does not allow a general conclusion for all esophageal cancers to be drawn; on the other hand, our data were not subject to variability due to biologically different cancers in different localizations.

We were confronted with a low number of lymph nodes differentiated in CT scans. Although this observation is not new in the literature [8, 18], the count of identified nodes was below our expectation. FDG-PET/CT examinations were carried out at high quality, carefully checked for high degree of standardization [24] according to PERCIST 1.0 protocol and fulfilled current EANM recommendations [22]. We did not find a way to improve FDG-PET/CT scans technically by changing the parameter settings.

The use of more than one radiologist would increase the reliability of the analysis from the statistical point of view. With a respect to the anticipated low sensitivity and high specificity, we dismissed it as a redundant intent. Even if a substantially better reader had made the double-reading, the increased sensitivity would have been from clinical point of view still unacceptably low and specificity would have remained still excellent. On the other hand, when a substantially worse reader had made the same, specificity would have dropped slightly, but it would have been probably still very good, and sensitivity would have remained unacceptably low.

A cogent reason for the use of high-resolution imaging (i.e., endoscopic ultrasonography) in nodal staging is the fact that more small lymph nodes can be imaged in the vicinity of the probe. Such a method could improve the ratio of imaged/removed lymph nodes and increase sensitivity  [30, 31]. That is why we tested whether increased sensitivity based on imaging of smaller objects would be also able to improve accuracy in the search for metastases. We set the threshold for the presence of metastases down when medium-large nodes 0.5–1.0 cm were also taken for positive. Using the threshold sensitivity increased 7.5 times compared to the single anatomic parameter. But, an increase of sensitivity was accompanied by a decrease in specificity of 20% and accuracy of 15%.

The next weak point in the diagnostics was the inability of FDG uptake imaging to prove metastases in imaged, but nonhypermetabolic nodes [28]. In our study, the parameter of hypermetabolism proved higher accuracy than node size. Unfortunately, the metabolic information was not sensitive enough in these diagnostics, but on the other hand was trustworthy due to the small number of false-positive findings. PET quantification using SUV is of special importance when measuring the effect of the treatment. We have used the PERCIST 1.0 in a longitudinal follow-up of lesions in the prospective study. In this paper, we have concentrated on a single-time point after chemotherapy and before surgery. In a single-time point scenario, utilization of SUV is of a limited value, when assessing relatively small lymph nodes, where the partial volume effect artificially lowers the response of the scanner. In this situation, a subjective assessment is more appropriate in deciding whether the uptake has increased above that of the adjacent soft tissue. Therefore we decided for this type of assessment.

**Fig. 1:**
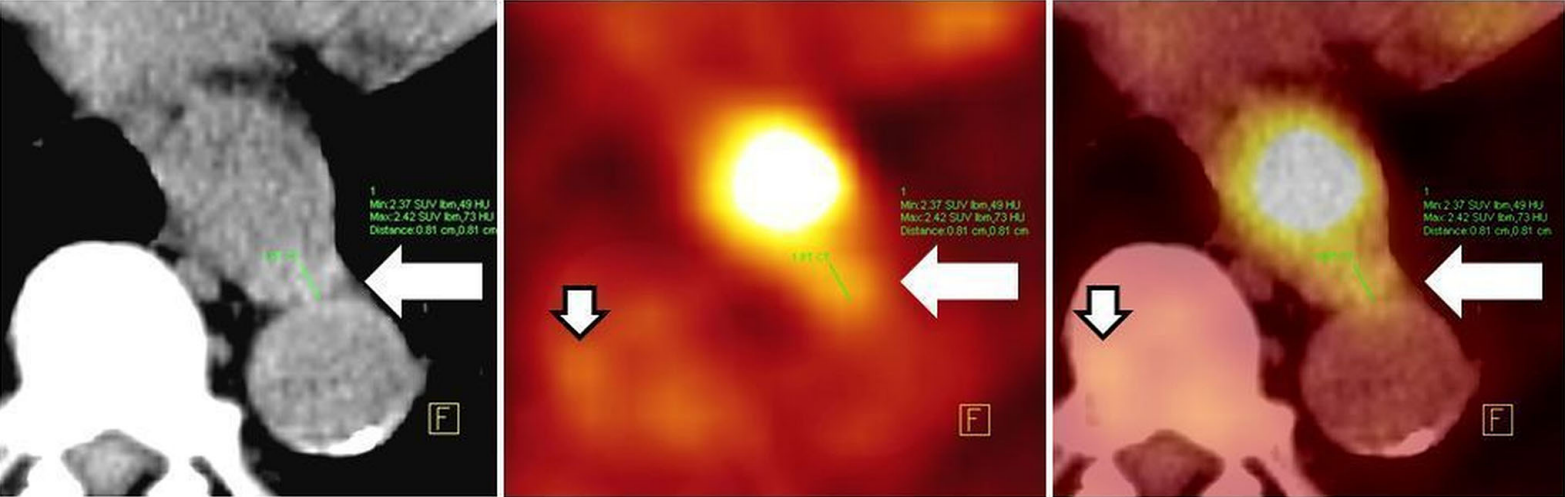
FDG-PET/CT imaging of small hypermetabolic lymph node. **A** (CT picture): small node with the short axis of 0.8 cm (*arrow*); **B** (FDG-PET picture): small hypermetabolic spot near to esophageal cancer, three similar spots are in the vicinity (*small arrow*); **C** (FDG-PET/CT picture): fused image diagnosed metastatic lymph node (*arrow*), and physiologic glucose metabolism in the bone marrow

FDG-PET/CT is a hybrid diagnostic tool where metabolic information provided by PET enhances the anatomic one provided by CT and vice versa. Without hybrid imaging, the reader would not be able to localize small hypermetabolic spots to the lymph node [14, 32] and differentiate small LNs as metastatic ones (Fig. 1). The simultaneous (“hybrid”) reading of both modalities is a robust tool in a nodal staging in routine practice, despite its low sensitivity.

As a result of our examinations, surgeons looked pointedly for lymph nodes with excessive metabolism, where metastases were present at 98.6%. Unfortunately it only occurred in 11.6% of regions. In other regions, surgeons sampled nodes blindly according to the standard protocol.

The low detection rate of removed and imaged LNs suggested the imaging with higher resolution should be used for lymph nodes identification. But the only anatomic improvement of imaging methods will probably be devalued by an increase of false positivity. The data analyzed in the study indicated that to reach higher accuracy, a higher resolution in lymph nodes imaging should be accompanied by an improvement of other nonanatomic parameters whether metabolic (new radiopharmaceuticals), molecular (MR spectroscopy, DWI), or others. The identification of smaller lymph nodes alone does not improve the accuracy.

## Conclusion

In a homogenous group of 74 patients with adenocarcinoma of the esophageal–gastric junction, nodal staging by FDG-PET/CT was not accurate due to the inability of FDG-PET/CT to image all lymph nodes and to verify micrometastases or metastases in small lymph nodes. The parameter “hypermetabolic nodes” proved to be of low sensitivity, but high specificity due to the low number of false-positive findings. Inflammation in lymph nodes mimicking metastases played a marginal role in this group of patients. The parameters “large nodes”, “large-and-medium large nodes”, and “large or hypermetabolic nodes” did not improve accuracy for low sensitivity, low specificity, or both. Due to low sensitivity, negative FDG-PET/CT findings could not exclude the presence of metastases; high specificity indicates that any positive nodal staging must be carefully considered.

## Abbreviations

R0: Radical resection of the cancer
FDG: 18F-fluoro-deoxy-glucose
SCC: Squamous cell carcinoma (of the esophagus)
AC: Adenocarcinoma (of the esophagus)
PERCIST 1.0: PET response criteria in solid tumors, version 1.0
EUS: Endoscopic ultrasonography
